# The dopaminergic system of *Caenorhabditis elegans*

**DOI:** 10.1098/rsos.250843

**Published:** 2025-10-22

**Authors:** Inchara Muralidhara, Iris Hardege

**Affiliations:** ^1^ Department of Zoology, University of Cambridge, Cambridge CB2 3EJ, UK

**Keywords:** dopamine, *C. elegans*, behaviour, model organisms, neuroscience

## Abstract

Dopamine is a highly conserved neurotransmitter that plays a pivotal role in regulating a wide array of behaviours. In vertebrates, it is best known for its involvement in motor control, motivation, reward processing and learning. Dysregulation of dopaminergic signalling is implicated in several human neurological and psychiatric disorders, most notably Parkinson’s disease. The fundamental importance of dopamine has driven researchers to study it across a range of model organisms. Among these, the nematode *Caenorhabditis elegans* has proven particularly valuable. With a compact and fully mapped nervous system, genetic tractability and transparent body, *C. elegans* provides a powerful system to unravel the mechanisms of dopamine synthesis, signalling, receptor function and behavioural modulation. Like in mammals, dopamine is produced by a small number of neurons, yet it governs complex behaviours including locomotion, learning and responses to environmental cues. In this review, we explore the breadth of research on dopaminergic signalling in *C. elegans*, focusing on its synthesis, receptor signalling and downstream effects on behaviour. By integrating findings across molecular, cellular and circuit levels, we aim to highlight both the conserved features of dopamine signalling and the unique insights gained from studying it in this model organism.

## 1. Introduction

Across the animal kingdom, dopamine, despite being produced by a relatively small number of neurons, influences a wide range of behavioural outputs from basic locomotion through to complex learning paradigms. In humans, dopamine plays a wide-ranging role in regulating movement, motivation, reward learning, executive function and mood [1–5]. Its dysfunction is central to several neuropsychiatric and neurodegenerative conditions, including Parkinson’s disease (PD), schizophrenia, depression and addiction [6–10]. These functions are largely mediated through distinct dopaminergic pathways originating from midbrain regions such as the substantia nigra and ventral tegmental area. While the brain structures involved are often much simpler, animals from vertebrates through to invertebrates also use dopamine signalling in similar ways and have long been employed as models for studying dopaminergic behaviours.

One such organism is the nematode *Caenorhabditis elegans* (*C. elegans*), which despite possessing a nervous system composed of only 302 neurons also uses dopamine as a neurotransmitter for a wide range of behaviours from locomotion through to egg laying and learning [11]. *Caenorhabditis elegans* is a widely used model organism in neuroscience because of its comprehensively mapped genome and nervous system [12] as well as the surprising level of conservation of key neurobiological mechanisms including synaptic communication and neural development [13]. The animal’s compact nervous system allows for direct links to be drawn between molecular components of dopamine signalling, specific neurons and circuits and well-characterized behaviours and has been instrumental in dissecting mechanisms of dopaminergic cell identity [14], neurodegeneration [15] and behavioural control [16]. Despite the many similarities between nematode and vertebrate dopaminergic systems, there are also key differences, including the presence of fast-acting ion channel receptors gated by dopamine [17,18], which are present only in invertebrates and offer an opportunity to study mechanisms governing synaptic and extrasynaptic signalling at the molecular level.

Here, we focus on the non-pathological aspects of dopamine signalling*;* however, *C. elegans* has also proved to be an invaluable model organism in deciphering the molecular mechanisms of ageing and dopaminergic neurodegeneration. Several *C. elegans* models of PD have been developed, including toxin-induced (6-hydroxydopamine) and genetic models expressing human genes such as α-synuclein and LRRK2 [15,19–23]. These models have been essential for identifying neuroprotective pathways, such as autophagy and antioxidant systems [24], and for high-throughput screening of potential PD therapeutics [25] and have been reviewed thoroughly [26–28]. In ageing studies, dopamine has also been implicated in food-induced lifespan reduction [29,30].

In this review, we examine our current understanding of the dopaminergic system of *C. elegans* in detail, focusing on the synthesis, release and action of dopamine, and the behaviours it modulates. We also consider and discuss the surprising breadth of questions that remain within this long-studied area.

## 2. Dopamine synthesis, release and metabolism

In 1975, shortly after *C. elegans* was established as a model organism, the use of dopamine in the nervous system was described by Sulston and Brenner using chemical staining techniques [11]. This work identified the eight dopamine-producing neurons (CEPs, PDEs and ADEs), with a further six dopaminergic ray neurons (R5A, R7A and R9A) found specifically in males. These findings have since been confirmed by several studies, most recently using CRISPR-Cas9-based reporters to map the expression of key dopamine synthesis and release machinery [31]. The male-specific non-neuronal socket cells have also been shown to express dopamine biosynthesis pathway genes [32].

### 2.1. The dopaminergic neurons

Dopaminergic neuron fate is controlled by a simple conserved *cis*-regulatory element called the dopamine motif that leads to the expression of dopamine synthesis pathway genes and is activated by the ETS (Erythroblast Transformation Specific) family transcription factor *ast‐1*. Animals lacking *ast‐1* lose the ability to generate the dopaminergic neurons, while overexpression of *ast-1* can drive dopaminergic cell identity [14]. Interestingly, the mouse homologue of *ast‐1*, Etv1 [33], could also rescue the *ast‐1* phenotype in *C. elegans* [14], highlighting the conservation of this pathway across animals.

**Figure 1.**
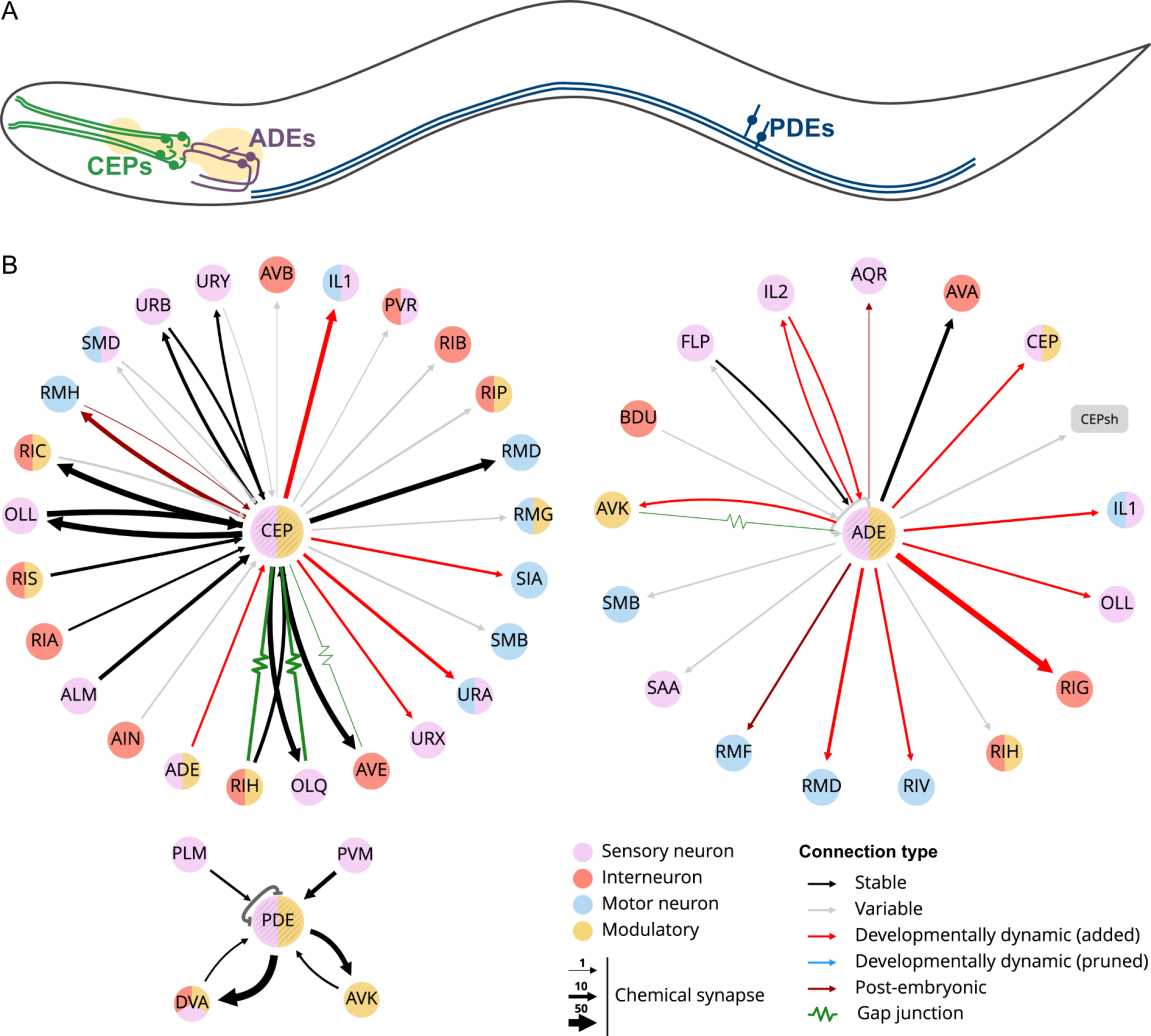
Position, morphology and connections of *C. elegans* dopaminergic neurons in the hermaphrodite. (A) Representative position and morphology of dopaminergic neurons. Pale yellow represents the position of the pharynx. The worm is orientated with the anterior to the left, the posterior to the right and the ventral side at the bottom. Both the left and right neurons are shown in their approximate positions. (B) Connectivity maps of the dopaminergic neurons, generated with NemaNode [34] using data from [12,34,35].

All three sets of dopaminergic neurons have ciliated endings that terminate in sensilla throughout the body of the worm. These sensilla are simple sense organs composed of one or more neuronal dendrites surrounded by sheath and socket cells ([12]; figure 1A). The four CEPs (cephalic sensilla neurons) consist of two pairs of dorsal and ventral cells (CEPDL/R & CEPVL/R), which are located close to the nerve ring. Each cell sends anterior processes to the tip of the nose where they end in the cephalic sensilla and posterior processes into the nerve ring. The main synaptic outputs from the CEPs are the octopaminergic neuron RIC, the backward locomotion-tuned interneuron AVE, the sensory neurons OLL and OLQ and the motor neuron RMD, as well as a variety of other head neurons (figure 1B). The CEPs also have gap junctions with OLQ and RIH and receive some synaptic input from OLL and a variety of other neurons ([12,34,35]; figure 1B). The pair of ADE neurons (anterior deirid neurons) is located behind the posterior bulb of the pharynx, with anterior processes entering the anterior dendrid sensilla located on the alae, the protruding longitudinal ridge on the surface of the worm (figure 1A). The dorsal ADE processes extend into the nerve ring, where their major synaptic outputs are onto the backwards locomotion-tuned interneuron AVA and the interneuron RIG, among others, including the CEPs ([12,34,35]; figure 1B). The ADEs, however, receive little synaptic input with a low and variable number of synapses identified from the mechanosensory neurons FLP and IL2 ([12,34,35]; figure 1B). Finally, the pair of PDE neurons (posterior deirid neurons) is positioned sub-ventrally in the posterior body, with their dorsal processes ending in the posterior deirid sensilla that lie halfway between the vulva and the tail next to the body wall muscles. The ventral processes enter the central nerve cord and bifurcate in both the anterior and posterior directions to synapse onto the stretch receptor neuron DVA in the tail and sensory neuron ASK in the head (figure 1). The PDEs also receive significant synaptic input from the touch receptor neuron PVM ([12]; figure 1B).

### 2.2. Dopamine biosynthesis and release

**Figure 2.**
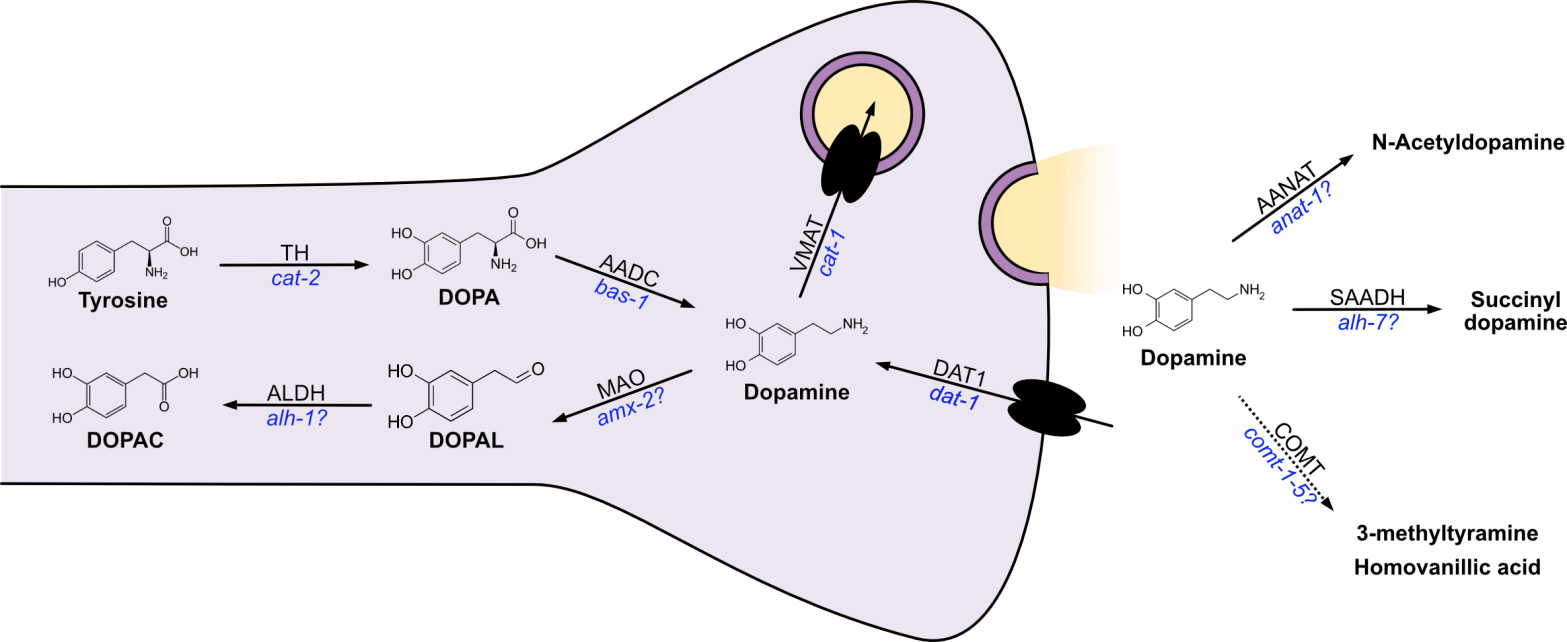
Dopamine synthesis, release and metabolism. Proposed *C. elegans* pathways for dopamine biosynthesis and metabolism in dopaminergic neurons. *Caenorhabditis elegans* gene names are shown in blue. The exact cellular/extracellular location in which dopamine metabolism takes place is not known.

Dopamine biosynthesis from the amino acid tyrosine is highly conserved across animals. It occurs via two key enzymatic reactions; tyrosine hydroxylase (TH) converts l-tyrosine to l-Dopa, which is decarboxylated to form dopamine via an aromatic l-amino acid decarboxylase (AADC; [36]; figure 2). *Caenorhabditis elegans* encode a single gene for TH called *cat-2* and several AADCs, with the most important and well-studied being encoded by *bas-1* [37]. They also encode a guanosine triphosphate (GTP) cyclohydrolase, *cat-4*, which is required for the biosynthesis of tetrahydrobiopterin, a cofactor of TH [16]. In neurons once produced, dopamine is packaged into synaptic vesicles via the vesicular monoamine transporter (VMAT) encoded by *cat1* [38]; its reuptake is via the dopamine transporter encoded by *dat-1* [39]. Mutant animals lacking *cat-1*, *cat-2* and *cat-4* have significantly reduced dopamine levels measured by biochemical means [11]. Despite animals with *cat-2* mutations showing only a partial reduction in dopamine levels, no alternative biosynthesis pathways have been identified in *C. elegans*, however, several uncharacterized AADCs exist, and an alternative serotonin synthesis pathway was recently described via acetylated intermediates [40]. There is also evidence for dopamine synthesis by the microbiome in other species [41]. It is also worth noting that multiple independent studies have not been able to detect the presence of the other catecholamines, norepinephrine and epinephrine, in *C. elegans* despite having overlapping biosynthesis pathways [11,42].

### 2.3. Dopamine metabolism

Dopamine metabolism pathways in *C. elegans* have not been extensively mapped, however, *C. elegans* contains several genes with homology to known pathways including monoamine oxidase (MAO) and catechol-O-methyltransferase (COMT) pathways, which are summarized in table 1. Animals with mutations in the MAO homologue *amx-2,* which has the highest similarity to mammalian MAOs, have been shown to exhibit higher serotonin concentrations, confirming its role as an MAO enzyme [44]; however, direct evidence for AMX-2 acting on dopamine is lacking. *Caenorhabditis elegans* also encode five homologues for COMT, *comt-1-5* [13], none of which have been functionally characterized, and their homology to mammalian COMTs is low (up to 30% identity). Behavioural evidence suggests COMT-4 may act on dopamine, as animals with high expression of *comt-4* show changes in dopamine-related behaviours [46]. An alternative metabolism and inactivation pathway via the acetylation of dopamine and other amines has been described in invertebrates, including nematodes, with *anat-1* encoding a putative arylalkylamine N-acetyltransferase (AA-NAT) in *C. elegans* [47]. High-performance liquid chromatography studies have revealed high levels of N-acetyldopamine as well as succinylated dopamine, which may represent another metabolism pathway [48].

**Table 1.** Overview of the genes involved in *C. elegans* dopamine synthesis, transport and metabolism and their human homologues.

function	human homologue	*C. elegans* homologue	expression
tyrosine hydroxylase (TH)	TH	*cat-2*	CEP, ADE, PDE [31]
aromatic l-amino acid decarboxylase (AADC)	DOPA	*bas-1*	CEP, ADE, PDE, HSN, NSM, ADF [31]
vesicular monoamine transporter (VMAT)	VMAT2	*cat-1*	CEP, ADE, PDE, NSM, RIM, HSN, VC4/5, RIC, ADF, CAN, RIH [31]
GTP cyclohydrolase	GTPCH	*cat-4*	CEP, ADE, PDE, HSN, NSM, ADF [43]
dopamine transporter	SLC6A3 (DAT1)	*dat-1*	CEP, ADE, PDE [43]
catechol-O-methyltransferase (COMT)	COMT	*comt-1-5*	broad expression across tissues [43]
monoamine oxidase (MAO)	MAOA	*amx-2*	I1, I2, I3, I4, NSM, M3, M5, RIC, AVA, AVG, glia, pharyngeal muscle [43,44]
aldehyde dehydrogenase (ALDH)	ALDH1B1	*alh-1*	muscle, intestine, epidermis, neurons [45]
arylalkylamine N-acetyltransferase (AA-NAT)	AANAT	*anat-1*	broad expression across tissues [43]
succinic semialdehyde dehydrogenase (SSADH)	SSADH	*alh-7*	broad expression across tissues [43]

## 3. Dopamine receptors

Like other animals, *C. elegans* express a range of dopamine receptors that have both activating and inhibitory effects on expressing cells and neurons. However, uniquely, nematodes also express fast-acting ligand-gated ion channel (LGIC) receptors gated by dopamine in addition to the traditional dopamine-activated G-protein-coupled receptors (GPCRs) found across the animal kingdom. In total, the *C. elegans* genome encodes eight known dopamine receptors, four GPCRs (*dop-1-4*) [49–51] and four LGICs (*lgc-51-54*) [17,18]. An overview of dopamine receptors from *C. elegans* is shown in table 2. In addition to known receptors, there are further receptors whose secondary ligand is dopamine, or which have been predicted from sequence homology. These are summarized in table 3. Receptors range in their affinity for dopamine, with most of the dop-GPCRs showing E_C_50 values in the nanomolar range (10 nM–10 µM), whereas the ion channel receptors have E_C_50s typically in the micromolar range (0.5−16 µM). This disparity in affinity is typical for receptors acting via alternative signalling strategies, with synaptic receptors often displaying higher E_C_50 values than extrasynaptic receptors. This suggests that both synaptic and extrasynaptic dopamine signalling may be employed in the nematode nervous system.

**Table 2.** Overview of known *C. elegans* dopamine receptors. (E_C_50 for dopamine, ion/G-protein assignment and dose response measured with two-electrode voltage clamp (TEVC) in *Xenopus* oocytes unless otherwise specified in notes. For simplicity, norepinephrine and epinephrine E_C_50s are not included owing to the lack of these transmitters in *C. elegans*.)

gene	type	ion/G-protein	E_C_50 for dopamine	E_C_50 for other amines	notes/references
*dop-1*	GPCR	Gs	10 nM	none	[42,49]
*dop-2*	GPCR	Gi/o	70 nM	not tested	E_C_50 by cAMP inhibition in CHO cells [50]
*dop-3*	GPCR	Gi/o	30 nM	tyramine 500 nM octopamine 250 μM	E_C_50 by cAMP inhibition in CHO cells [51]
*dop-4*	GPCR	Gs	10 μM	none	E_C_50 by cAMP accumulation in HEK cells [51]
*lgc-51*	LGIC	anion	0.8 μM	tyramine 4 μM	No current alone, heteromer with *lgc-52* [18]
*lgc-52*	LGIC	anion	0.5 μM	tyramine 54 μM	[18]
*lgc-53*	LGIC	anion	4 μM	none	[17]
*lgc-54*	LGIC	anion	16 μM	tyramine 13 μM 5-HT 94 μM	[18]

**Table 3.** Overview of other potential dopamine receptors. (E_C_50 for dopamine, ion/G-protein assignment and dose response measured with TEVC in *Xenopus* oocytes unless otherwise specified in notes.)

gene	type	ion/G-protein	E_C_50 for dopamine	E_C_50 for other amines	notes/references
*lgc-55*	LGIC	anion	160 μM	tyramine 12 μM	[52]
*lgc-56*	LGIaC	anion	640 μM	tyramine 70 μM	formerly *ggr-3* [18]
*dop-5*	GPCR	?	?	?	predicted from sequence homology [53]
*dop-6*	GPCR	?	?	?	predicted from sequence homology [53]

### 3.1. G-protein-coupled receptor-mediated neurotransmitter signalling

Nematode nervous systems employ GPCRs to mediate signalling via small-molecule neurotransmitters, which are thought to act predominately extrasynaptically and typically operate at longer time scales than transmission via ion channel receptors. The *C. elegans* genome encodes 1300 genes thought to encode GPCRs [54], among these are a group of 27 genes encoding neurotransmitter-activated GPCRs for acetylcholine, glutamate, GABA, serotonin, dopamine, tyramine and octopamine [55]. These receptors have mainly been shown to act via the three traditional G-protein signalling pathways: Gq (intracellular Ca^2+^ release), Gi/o (cAMP inhibition) and Gs (cAMP activation). However, *C. elegans* also encodes additional nematode-specific G-proteins that have narrow expression patterns and are not well studied [55]. Owing to the expression pattern of *C. elegans* neurotransmitter GPCRs, they are thought to act predominately via extrasynaptic signalling [56], however, recent studies in mammals have started to challenge this view, suggesting that certain dopamine functions necessitate spatiotemporal precision indicative of peri-synaptic or synaptic localization of dopamine receptors [57]. *Caenorhabditis elegans* express four well-characterized dopamine-activated GPCRs (*dop-1-4*) [49–51] and a further two putative receptors *dop-5-6*, which are predicted to be dopamine-activated based upon homology [53].

Functional characterization of GPCRs is typically carried out in heterologous expression systems, either in cell lines, typically mammalian HEK or CHO cells, or in *Xenopus* oocytes [58]. A variety of techniques, including luminescent plate-based assays, radioligand binding and electrophysiology, can then be used to assess ligand affinities and G-protein coupling either through the use of endogenous G-proteins present in the heterologous expression system or by supplementing with wild-type or so-called promiscuous G-proteins [59]. In 2002, the first dopamine receptor from *C. elegans* was identified by Suo and colleagues, who successfully cloned *dop-1*, a homologue of the human D1 dopamine receptor [49]. By expressing DOP-1 in the mammalian cell line COS-7 and using radioligand binding assays, they determined dopamine to be the primary ligand, with lesser binding by norepinephrine and other monoamines. Subsequently, Sanyal *et al.* performed an in-depth characterization in both COS-7 cells and *Xenopus* oocytes using radioligand binding and electrophysiology and found that DOP-1 couples to Gαs subunits, cAMP production and activation of the inwardly rectifying potassium channel Kir3.2 [42]. They also found dopamine to be the highest affinity ligand, with only norepinephrine and epinephrine of those ligands tested showing any receptor activation. Because *C. elegans* has been shown not to produce norepinephrine and epinephrine [11,42], this suggests that DOP-1 is a highly specific dopamine receptor. Three further dopamine- activated GPCRs were characterized shortly after, with *dop-2* and *dop-3* encoding D2-like receptors [50,51] and *dop-4* an additional D1-like receptor [51]. When expressed in mammalian cells, activation of both DOP-2 and DOP-3 by dopamine led to inhibition of cAMP, suggesting coupling to the inhibitory Gαi/o pathway [50,51], whereas activation of DOP-4 expressed in mammalian cells led to accumulation of cAMP as measured by a luciferase expression assay, suggesting coupling to Gαs [51].

### 3.2. Dopamine-gated ion channels

Despite the small size of the nematode nervous system, it employs a surprising variety of fast-acting neurotransmitter receptors activated by a broad range of chemical neurotransmitters [31]. Among these receptors are LGICs from the pentameric cys-loop family, which are gated by monoamines including dopamine. The cys-loop family also includes the classic GABA (GABA_A_Rs) and nicotinic acetylcholine receptors (nAChRs) that mediate fast synaptic transmission by passing either cations or anions to excite or inhibit post-synaptic cells. They may also be expressed pre-synaptically as auto receptors. *Caenorhabditis elegans* express six dopamine-gated LGICs (*lgc-51-56;* [17,18,52]), all of which come from the same subfamily of receptors, most closely related to vertebrate GABA_A_Rs, and like those receptors, they have been shown to pass anions and be inhibitory. Until recently, nematodes were the only animal known to use dopamine for fast neurotransmission, however, recent work has shown that other invertebrates, including molluscs and insects, express dopamine-gated receptors that are closely related to vertebrate nAChRs α9/10 [60] and therefore are only distantly related to the nematode dopamine LGICs. With many putative LGIC genes in *C. elegans* still uncharacterized, further dopamine receptors may yet be discovered.

Initial characterization of LGICs is typically carried out in *Xenopus* oocyte heterologous expression systems in which two-electrode voltage clamp (TEVC) is used to assess the electrophysical properties of ion channels, including ligand specificity and ion permeability. In 2009, Ringsted and colleagues used TEVC to identify and functionally characterize the first dopamine-activated LGIC, LGC-53 [17]. They found that LGC-53 formed an anion-selective homomeric channel gated specifically by dopamine with little activity in the presence of other monoamines, and that this dopamine-induced current could be blocked by a range of dopaminergic GPCR antagonists including spiperone. More recently, phylogenetic analyses revealed a subfamily of putative monoamine-gated channels [61] which were characterized using TEVC by Morud *et al.* [18]. This revealed a further three dopamine-gated LGICs (LGC-51, 52 and 54). Like LGC-53, these receptors were anionic, however, in contrast, they showed less specificity to dopamine. LGC-52 homomers displayed reasonable activation by tyramine, and LGC-54 homomers were activated to a similar extent by dopamine, tyramine and serotonin. LGC-51 was shown to form a heteromeric channel in combination with LGC-52, with the heteromeric form displaying relatively increased affinity for tyramine. Despite their co-reactivity to other monoamines, LGC-51, 52, 53 and 54 are for simplicity referred to as dopamine receptors, as their preferential ligand appears to be dopamine or their affinities for multiple ligands are equal. Two other members of this subfamily LGC-55 and LGC-56 encode tyramine receptors, which also display reasonable affinity for dopamine, although their primary ligand appears to be tyramine with a tenfold lower E_C_50 [18,52]. It is worth noting that the behavioural characterization of the dopamine-gated LGICs is sparse, and further work is needed to determine whether these receptors function as dopamine receptors *in vivo* within the nervous system.

### 3.3. Dopamine receptor expression

In nematodes, much like in vertebrate brains, dopamine is released from only a small proportion of the nervous system, but its receptors are broadly expressed, summarized in table 4. Our current understanding of dopamine receptor expression is based on published expression patterns, which are primarily based on over-expressed short promoter fusions and single-cell RNAseq [43]. The resulting expression patterns observed by reporter-based strategies and RNAseq do not 100% correlate, with broader expression observed by RNAseq for all receptors (table 4). Despite these discrepancies, for each receptor, there is a set of neuron classes that have been identified by both approaches. This reveals that both GPCR and LGIC receptors appear to be expressed in a range of sensory, inter and motor neurons, and both are expressed in neurons that receive synaptic dopamine input and those that do not. As previously mentioned, dopaminergic GPCRs are typically thought to act extrasynaptically and LGICs synaptically, partly owing to their expression pattern. A previous study in 2016 of the monoamine networks in *C. elegans* found that of the neurons expressing the dopamine receptors then known, *dop-1-6* and *lgc-53,* 82% of cells received no direct dopaminergic inputs [56], supporting the notion that dopamine-activated GPCRs act predominately extrasynaptically.

**Table 4.** Neuronal expression pattern of dopamine receptors in *C. elegans*. (The bold number at the start of the column is the total number of classes expressing this identified by this method (including those that overlap). RNAseq expression is set to threshold 2.)

gene	expression observed by reporters and RNAseq [43]	expression observed only in reporters	expression observed only by RNAseq [43]
*dop-1*	AUA, RIM, ALM, PLM, PHC, AVM, PVQ, RIS, PVD, VA, VB, AS, DA, DB [42,53,62]	**(17)** RIB, ALN, PLN [42,53,62],	**(63)** ADA, ADE, AIA, AIB, AIM, AIN, AIZ, ASE, ASI, ASJ, ASK, AVA, AVB, AVD, AVE, AVG, AVH, AVJ, AVK, AWC, BDU, DVC, FLP, I1, I3, I5, IL1, LUA, M5, PDB, PDE, PVM, PVN, PVP, PVR, PVT, PVW, RIA, RID, RIF, RIG, RIP, RIV, RMD, RMF, RMH, SMB, SMD, URB
*dop-2*	ADE, PDE, CEP, RID, RIA, SIB, SIA [50,62]	**(8)** PDA [50,62]	**(41)** ADF, ASH, ASK, AVA, AVG, AVH, AVK, AWA, AWC, DA, DA9, DB, DVA, DVC, IL2, OLQ, PDB, PVM, PVT, RIC, RIG, RIM, RIR, RIV, RMD, RME, RMF, SDQ, SMD, URB, VA, VB, VD/DD
*dop-3*	PVD, VA, VB, AS, DA, DB, DD, VD, ASE, RIC, SIA, NSM, ASK [53,63–66]	**(13)**	**(46)** ADF, AIM, ALA, ASH, AVA, AVB, AVD, AVE, AVF, AVG, AVK, AWA, AWB, CEP, DVB, FLP, I1, IL1, IL2, PHA, PHC, PLM, PVC, PVM, PVN, PVQ, RIF, RIG, RIM, RIS, RME, SAB
*dop-4*	I2 [51]	**(6)** AVL, ASG, PQR, I1, CAN [51]	**(23)** OLL, AIM, ALA, ALM, ASE, AVA, AVB, AVH, AVK, CAN, DA9, FLP, I3, I4, M1, NSM, PLM, PVM, RIC, RMH, SIA, SIB
*lgc-51*	RMD [18]	**(2)** SMD [18]	**(5)** AUA, AVB, PVC, RME
*lgc-52*	RMD, SAA, SMD [18]	**(3)**	**(5)** RME, URB
*lgc-53*	HSN, PVD, IL2, VA, FLP, AVF, URY [56]	**(9)** CAN, AIM [56]	**(43)** AVE, ADE, ALA, ASG, AVA, AVH, AVJ, AWA, BAG, BDU, CEP, DVA, DVC, LUA, OLL, PDE, PHC, PVM, PVR, PVW, RIB, RIC, RIG, RIH, RIM, RIP, RIS, RMF, RMG, RMH, SIA, SIB, SMD, URX, VB, VD/DD
*lgc-54*	AVE, RMH [18]	**(2)**	**(18)** RME, AIN, ASH, AVH, PDE, PVD, PVW, RIC, RIS, RMD, RME, RMF, RMG, SAB, URB, VA

The activating D1-like receptors *dop-1* and *dop-4* are broadly expressed in interneurons and motoneurons, with *dop-1* being particularly broadly expressed, with 63 neuron classes showing expression by RNAseq and 17 classes by reporter fusions. The D2-like inhibitory receptors *dop-2* and *dop-3* are also expressed in interneurons and motoneurons, *dop-2*, in particular, shows high levels of expression in the dopaminergic neurons themselves (by both RNAseq and reporter fusions), leading to *dop-2* being referred to as an autoreceptor, which it has been suggested plays a role in controlling dopamine release, similar to the role of mammalian D2 receptors [67,68]. The notion that inhibitory dopamine-gated ion channels act synaptically hinges on their expression in neurons that receive synaptic input. For *lgc-51*, *52* and *54,* there is clear expression in neurons that receive high levels of dopaminergic input including RMD and AVE, however, *lgc-53* has a much broader expression pattern, with many neurons receiving no direct dopamine synapses, including the touch receptor neurons and HSN. Interestingly, both GPCR and LGIC receptors are expressed extensively in mechanosensory neurons, including the touch receptor neurons and the harsh touch neurons PVD and FLP (*dop-1, dop-3, lgc-53*), highlighting a potentially complex role for dopamine in the regulation of mechanosensory behaviours. In addition to neuronal expression, GPCR dopamine receptors have been identified in non-neuronal tissue including body wall muscle and rectal glands [51,53]. *dop-1-3* are expressed throughout the egg-laying circuit, including *dop-1* expression in the neuroendocrine uv1 cells that are involved in controlling egg laying [69,70]. In males, *dop-4* has been shown to be expressed in the male-specific RA8 neurons [51] and *dop-2* in unidentified male-specific tail neurons [50]; however, the expression of dopamine receptors in males more generally is not well characterized.

The broad and complex expression pattern of dopamine receptors in the *C. elegans* nervous system underlies the breadth of behaviours in which dopamine plays a role; these are addressed in further detail in the rest of this review.

## 4. Locomotion regulation by dopamine

The role of dopamine in controlling locomotion is well characterized across the animal kingdom, with deficiencies in dopamine signalling leading to PD in humans, a debilitating locomotive disorder [9,10]. In *C. elegans*, locomotion defects are also a hallmark of deficiencies in the dopaminergic system. Locomotion in *C. elegans* is characterized by two main types of movement—swimming and crawling—each with distinct features and underlying neural mechanisms [71]. Crawling occurs on a solid surface and involves generating sinusoidal waves along the length of the body to generate an S-shaped conformation. Waves occur at an undulation frequency of approximately 0.5 Hz. When placed in a liquid medium, *C. elegans* exhibit swimming (previously known as thrashing) behaviour, with the lower mechanical load of the liquid medium inducing a C-shaped conformation. Worms swim at a higher frequency around 2 Hz [72].

On solid surfaces, there are several basic patterns of crawling locomotion in *C. elegans*, consisting of forward and backward movement, and two types of turns: omega and reversals. The primary form of locomotion is forward movement, which is often interrupted by turns. In an omega turn, the head curls back, touching or crossing the tail while the worm is still moving forward (resembling the Greek letter omega (Ω)) and typically occurs on the ventral side of the body. A reversal occurs when a worm shifts from forward to backward movement without the head curling back [73]. These basic locomotive patterns are modulated by several factors, including the feeding state of the animal, temperature and the presence or absence of food. Among these, dopamine modulates locomotion across various contexts like altered movement [53,74,75], basal slowing response (BSR) [16], transitions between swimming and crawling [76,77], fine-tuning motor activity [78] and motor programme coupling [79]. These roles of dopamine in controlling locomotion are described in detail below.

### 4.1. General locomotion

**Figure 3.**
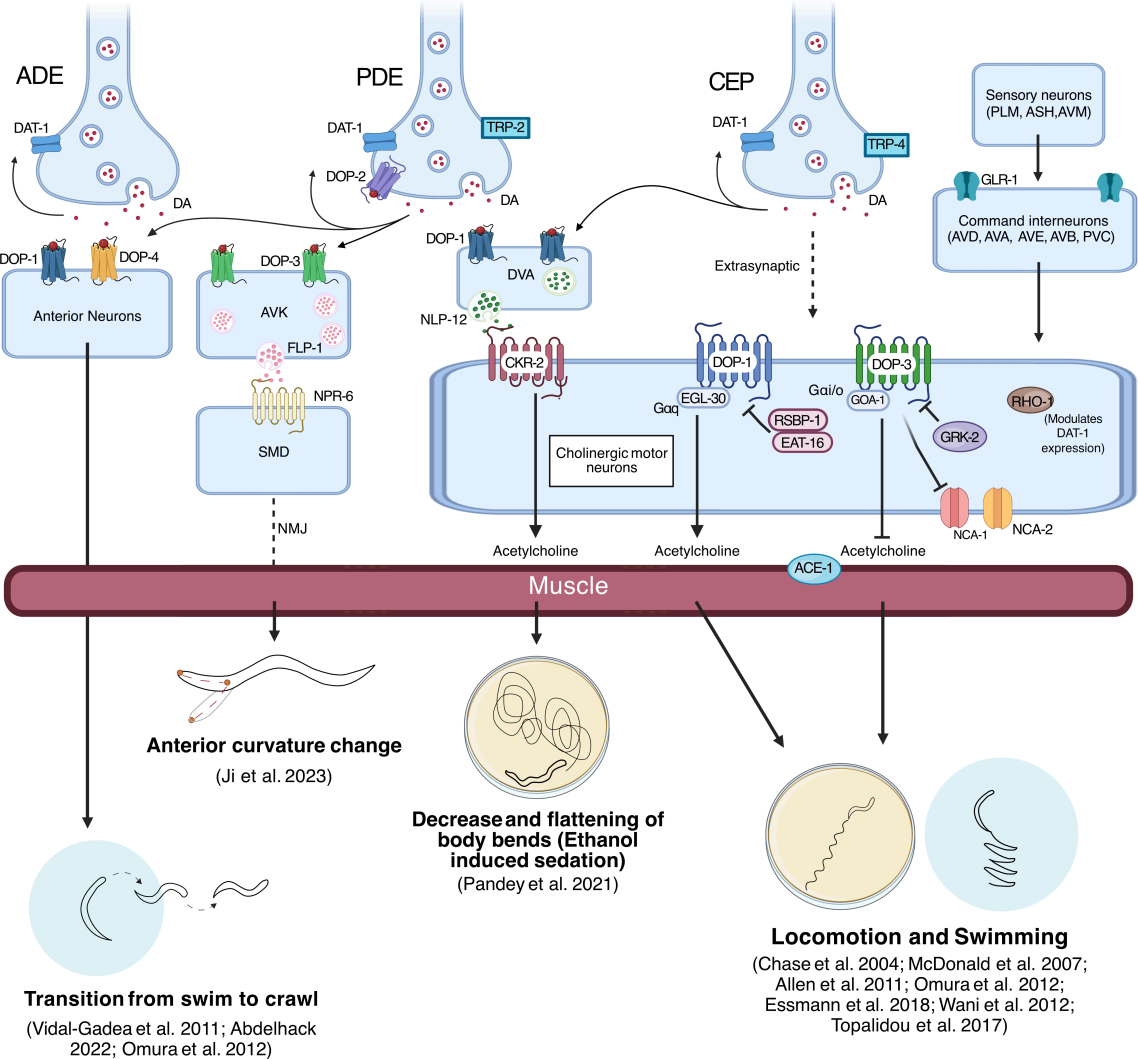
Overview of dopaminergic neural pathways and molecular mechanisms contributing to locomotion behaviours in *C. elegans*. Dopamine (DA) is released from ADE, CEP and PDE neurons and acts on dopamine-activated GPCRs expressed in a range of neurons, including AVK, DVA and cholinergic motor neurons, to influence muscle activity and lead to changes in locomotion. In addition, dopamine signalling is also modulated by GLR-1 expressing interneurons. Created in BioRender. Muralidhara, I. (2025) (https://BioRender.com/3ji3obg).

Dopamine alters the precise rate of locomotion through multiple interconnected molecular and cellular mechanisms (figure 3). The D2-like receptor, DOP-3 and its G-protein subunit GOA-1 are essential for *C. elegans* to make minor adjustments in their speed, which help them maintain a constant locomotion rate. Mutants defective in dopamine synthesis exhibit highly variable locomotion rates, characterized by frequent shifts between abnormally low and high speeds, along with altered body bend amplitude indicating that dopamine normally acts to stabilize movement [78]. Central to dopamine’s regulatory function is the antagonistic action of two distinct receptor types in the cholinergic motor neurons [53]. The D1-like receptor, DOP-1, coupled to Gαq subunit EGL-30 promotes locomotion by enhancing acetylcholine (ACh) release, while the D2-like receptor, DOP-3, inhibits movement by suppressing ACh release through coupling with the Gαi/o, GOA-1 (figure 3; [53,75]). This dual receptor system provides a mechanism for the bidirectional control of locomotion.

The regulation of dopamine signalling is controlled by multiple factors, including the dopamine transporter DAT-1, which removes dopamine from the synaptic cleft and reduces synaptic dopamine spillover [74] and the expression of its receptors. Non-autonomous RHO-1 (RhoA orthologue) signalling from cholinergic motor neurons has been shown to decrease DAT-1 expression in dopaminergic neurons, thereby affecting dopamine signalling intensity [80]. EAT-16, a regulator of G-protein signalling (RGS) and RSBP-1, a membrane-targeting subunit required by Gαq, act together to inhibit DOP-1 signalling in cholinergic motor neurons to modulate locomotion (figure 3; [81]). This regulatory network is further complicated by HLH-17, a transcription factor expressed in the cephalic sheath cells supporting the CEP dopaminergic neurons. HLH-17 regulates the expression of the dopamine receptors DOP-1, DOP-2, DOP-3, the RGS protein EGL-10 and DAT-1 [82,83], thereby modulating locomotion. Dopamine also interacts with non-selective cation channels NCA-1 and NCA-2 as well as GRK-2 (a G-protein-coupled receptor kinase), by signalling via DOP-3 receptors to negatively regulate activity (figure 3; [84]).

The mechanistic basis of dopamine’s effects on locomotion features an important proprioceptive feedback system called the compensatory curvature response. In this pathway, dopaminergic PDE neurons detect midbody curvature through TRP-2 channels and modulate anterior bending through a defined circuit: dopamine acts on DOP-3 receptors in AVK interneurons, which release FLP-1 neuropeptides to activate NPR-6 receptors on SMB head motor neurons (figure 3). This pathway optimizes locomotor efficiency by enabling appropriate bending amplitudes across different environmental conditions [85].

Beyond its direct effects on locomotion, dopamine plays a crucial role in behavioural integration, coordinating movement with other activities such as egg laying. This is particularly evident in the coupling of egg-laying behaviour with roaming states, where dopamine and the D2-like receptor DOP-3 are required for maintaining appropriate egg-laying rates during periods of exploration [79].

### 4.2. Swimming-induced paralysis

During prolonged bouts of swimming, excess levels of dopamine caused by loss of function mutations in the dopamine transporter *dat-1* lead to a phenomenon called swimming-induced paralysis (SWIP). In this paradigm, *dat-1* mutant animals experience motor paralysis after approximately 10 min of swimming, which is not seen in wild-type animals or in animals lacking both dopamine synthesis and reuptake (*cat-2* and *dat-1*), suggesting that paralysis is caused by the accumulation of dopamine [74].

Both DOP-1 and DOP-3 receptors, which are expressed in cholinergic and GABAergic motor neurons, have been shown to mediate this behaviour [75]. The *dat-1* induced SWIP phenotype is suppressed by mutations in *dop-3*, suggesting that dopamine accumulation caused by a loss of *dat-1* leads to the activation of DOP-3, resulting in the inhibition of ACh release and consequently reducing muscle contraction (figure 3). While *dat-1/dop-1* double mutants do not show any rescue in swimming behaviour, triple mutants of *dat-1*, *dop-1* and *dop-3* showed an improved swimming rate in comparison with *dat-1/dop-1* double mutants, suggesting that DOP-1 may act to enhance ACh release and improve locomotion in the absence of DOP-3 [74,75].

In addition to dopamine receptor mutants, mutations in glutamate receptor and acetylcholinesterase encoding genes, *glr-1* and *ace-1*, also suppress the *dat-1* SWIP phenotype; both of these mutations are predicted to increase ACh signalling from the ventral nerve cord (figure 3; [75]). Several other proteins have also been found to modulate the SWIP phenotype, including EAT-16, RSBP-1, UNC-43, FLP-1 and GRK-1. RSBP-1 interacts with EAT-16, which function together in cholinergic motor neurons to specifically inhibit DOP-1 receptor signalling [81]. HLH-17 also influences SWIP, with quantitative polymerase chain reaction analysis in *hlh-17* mutants revealing reduced messenger RNA expression levels of *dat-1, dop-1* and *dop-3* [83]. Furthermore, mutations in *rnt-1*, which encodes a RUNX transcription factor, show a similar SWIP phenotype to *dat-1* mutants [86].

Forward genetic screens also revealed several novel alleles, *vt21*, *vt22*, *vt25* and *vt29,* which display a SWIP phenotype. Two are point mutations in *dat-1; vt21* encodes a missense mutation (G460D) affecting a highly conserved glycine residue, and *vt22* encodes a nonsense mutation (W283Stop), leading to a premature stop codon. The other two alleles, *vt25* and *vt29*, do not have mutations in *dat-1* but still exhibit the SWIP phenotype. These mutations are in different genomic regions that do not contain known dopamine signalling genes, suggesting that *vt25* and *vt29* represent novel genes involved in this pathway [87].

### 4.3. Transition from swimming to crawling

*Caenorhabditis elegans* use biogenic amines to switch between different forms of locomotion. When *C. elegans* encounter changes in environmental resistance, such as moving from a liquid to a solid surface, mechanosensory dopaminergic neurons—specifically the ADE and PDE neurons—detect these changes and release dopamine [78]. Dopamine then acts via the D1-like dopaminergic receptors DOP-1 and DOP-4 [77] to mediate the changes in locomotion required to transition from swimming to crawling (figure 3; [76]). By contrast, serotonin promotes the transition from crawling to swimming and suppresses crawling behaviours when worms are in a liquid medium. Therefore, the balance between dopamine and serotonin signalling is critical in regulating gait transitions in *C. elegans*, ensuring the animal adopts the appropriate locomotory pattern for its current environment [76].

### 4.4. Ethanol-induced sedative behaviours

The putative dopamine auto receptor DOP-2 plays an important role in regulating dopamine levels and downstream signalling pathways that modulate locomotion in response to ethanol exposure. When exposed to ethanol, mutants lacking DOP-2 display a unique ethanol-induced sedative(EIS) behaviour characterized by reduced and flattened body bends, particularly in the posterior region. This phenotype can be rescued by expressing DOP-2 in dopaminergic neurons, specifically in the posterior PDE neuron [67]. The authors propose a model where chronic ethanol exposure leads to increased dopamine release from the PDE neuron in *dop-2* mutants. This excess dopamine activates DOP-1 receptors on the DVA interneuron, leading to increased release of the neuropeptide NLP-12. NLP-12 then acts on CKR-2 receptors on cholinergic motor neurons, potentially enhancing ACh release at neuromuscular junctions(NMJs) (figure 3). This heightened cholinergic signalling results in the observed EIS behaviour, characterized by reduced and flattened body bends.

## 5. Feeding and foraging behaviours

Foraging behaviours are fundamental for survival across all organisms, enabling efficient food acquisition through complex neural mechanisms. In their natural habitat, the soil-dwelling *C. elegans* usually inhabits decaying plant matter rich in their bacterial food sources. These food sources can be highly variable in their bacterial content and availability, requiring them to optimize their ability to locate and make use of food sources efficiently [88]. In *C. elegans*, dopamine has been shown to integrate environmental cues with internal states to optimize foraging strategies, allowing worms to detect food and modulate related behaviours to maximize survival.

### 5.1. Basal slowing response

The basal slowing response (BSR) is a crucial adaptive behavioural response in *C. elegans* in which well-fed worms reduce their rate of locomotion upon entering a food source [16]. This response is primarily mediated by dopamine signalling, enabling effective foraging behaviour by allowing worms to remain within nutrient-rich environments. Mutations in any of the dopamine synthesis pathway genes (*cat-2, bas-1, cat-4*) display defects in this behaviour [16]. The initiation of BSR occurs through the mechanosensory activation of the CEP neurons, via the TRPN channel TRP-4, which is triggered by the physical contact between worm’s body and its food source (bacteria; figure 3; [89]). Specifically, the pair of dorsal CEPs (CEPD) shows a gradual activation upon entering bacterial lawns, whereas the ventral pair (CEPV) displays a quicker but weaker activation, and the posterior pair shows no activation [90]. The D2-like receptor, DOP-3, appears to be the main receptor responsible for the BSR behaviour, as animals lacking *dop-3* fail to slow down when entering a food source [53]. *dop-3* is highly expressed in the GABAergic and cholinergic motor neurons along the ventral nerve cord, where it is thought to function through coupling with the G-protein subunit GOA-1 [53,75], ultimately leading to a decreased ACh release from the ventral cord motor neurons. This reduction in ACh release results in decreased muscle contraction, manifesting as slower locomotion (figure 3; [75]). The remaining dopamine-activated GPCRs, DOP-1 and DOP-2, do not directly influence BSR [53].

Several other genes have also been associated with dopamine’s regulation of the BSR. Loss of the neuroligin NLG-1 leads to defects in BSR and to associated increases in expression of *comt-4,* a putative dopamine COMT enzyme that may lead to increased levels of dopamine degradation [46]. Mutations in the *C. elegans* homologue of CNTNAP (type 1 adhesion molecules), *nlr-1*, affect BSR by causing structural defects in the CEP neurons including axon fasciculation, increased distance between dopamine neuron axons, higher volume of axon projections and reduced CLA-1 puncta (a presynaptic active zone marker; [91]), as well as mislocalization of DOP-3.

### 5.2. Food search behaviour

When well-fed *C. elegans* are removed from food, they initially display high-angle turns, restricting themselves to searching a small area—a behaviour known as local search or area-restricted search (ARS). After approximately 30 min without food, worms transition to a global search strategy characterized by linear movements covering larger areas. Brief contact with food can reset this behaviour, indicating its responsiveness to food availability [92,93]. Exogenous application of dopamine leads to an increase in high-angled turns typical of ARS, which is blocked by the addition of the dopamine antagonist raclopride. In addition, animals in which the dopaminergic neurons have been ablated or dopamine synthesis is blocked through mutation of *cat-2* show a significant reduction in ARS [92]. Animals lacking glutamatergic signalling are also defective in ARS, and interestingly, the effect of mutations in the glutamatergic receptors *glr-1* and *glr-2* could not be rescued by exogenous dopamine, suggesting that dopamine acts upstream of glutamate signalling to regulate ARS behaviour [92]. Interestingly, the neuropeptide NLP-12 (cholecystokinin homologue) also modulates ARS via interaction with the DOP-1 receptor (figure 3), which may represent an evolutionarily conserved pathway, as similar interactions exist between dopamine and cholecystokinin in mammals [94].

**Figure 4.**
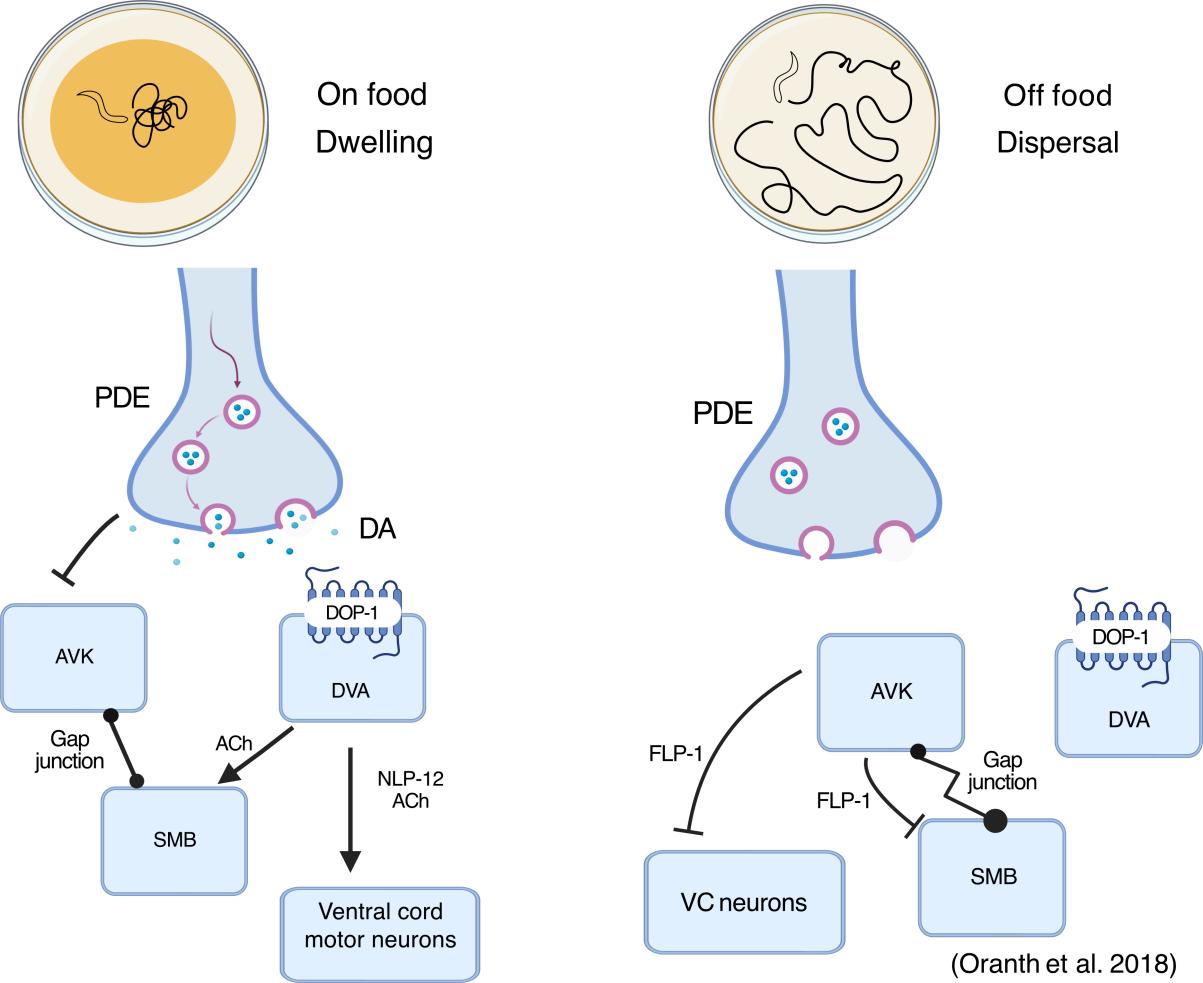
Dopaminergic regulation of foraging behaviours in *C. elegans*. Dopamine plays a key role in modulating the balance between dwelling and dispersal during foraging. In the presence of food, dopamine is released and acts on the DVA and AVK interneurons to promote dwelling behaviour. The transition to dispersal occurs during the absence of food, as the level of dopamine decreases, leading to the activation of the inhibitory action of AVK on VC neurons. Created in BioRender. Muralidhara, I. (2025) (https://BioRender.com/3ji3obg).

In the presence of food, high levels of dopamine act to suppress locomotion by inhibiting RIC and SIA neurons that express inhibitory DOP-3 and DOP-2 receptors, respectively [64,95,96]. This leads to a reduction of octopamine release from RIC and reduces ACh release from SIA, leading to decreased locomotor activity seen on food. In addition, dopamine also activates the DVA neurons that promote dwelling behaviour (figure 4; [97]). Conversely, in the absence of food, the lack of inhibition of RIC neurons leads to octopamine release, which is associated with starvation-like behaviours [64,95,96,98]. A reduction in dopamine signalling is also thought to allow tonic activation of AVK that releases the neuropeptide FLP-1, which inhibits SMB and VC motoneurons, thereby promoting dispersal behaviour (figure 4; [97]).

Interestingly, once animals have identified food sources, dopamine also appears to be important in making value-based decisions on the quality of different food sources, with *cat-2* mutant animals being defective in making consistent choices based on food quality [99].

### 5.3. Modulation of food search behaviours by internal and external states

Plasticity in food search behaviours is important for animals to navigate their complex environments; these can be driven by both internal and external factors. For example, worms modify their search strategies based on the size of previously encountered bacterial patches, specifically by adjusting large angle turns in ARS. Thus, animals removed from a large food patch show a 20% increase in their turning rate compared with animals removed from a small food patch. This plasticity involves the sensory neurons ASI and ASK, which show higher activity in high variability environments [100]. Like animals with ablated ASI and ASK neurons, *dat-1* and *cat-2* mutant animals also do not show changes in ARS depending on previous food environments, suggesting dopamine plays a role in regulating this behaviour, probably via the synaptic connection between ASK and CEP. In addition, *dop-1, dop-3* and *dop-4* mutants are also defective in this behaviour, with DOP-1 acting in ASI, probably increasing its activity and DOP-4 acting in the interneurons AIB and AIZ, whose activity is crucial to the animal’s ability to perform ARS and chemotaxis, respectively [100].

Internal states, in particular metabolic states, can significantly impact food search [93]. A dopamine-mediated example of this can be seen in dauer animals, a larval stage brought about by extended periods of food deprivation. Dauers exhibit a lower baseline locomotor activity, which can be increased both by increases and decreases in dopamine signalling, through loss of *dat-1* or *cat-2* [101]. This is contrary to normal adult animals that display decreases in locomotion with increased dopamine signalling, as seen in BSR and SWIP. These changes require DOP-3 and are probably owing to transcriptional changes via the transcription factor DAF-16 [101].

When animals are faced with both potential food sources and external dangers, they must modulate their priorities to either favour food acquisition or escape. The antagonistic action of D1 and D2-like dopamine receptors has been shown to be important for these context-specific changes in behaviour. For example, in experiments where animals must cross an aversive Cu^2+^ barrier to access an attractive odour (diacetyl), loss of *dop-1* led to an increased tendency to cross the barrier, while loss of *dop-2* or *dop-3* led to a decrease in crossing tendency [102], with DOP-1 probably acting in cholinergic neurons and DOP-3 acting in GABAergic neurons. Triple mutants of *dop-1-3* phenotypically copied *dop-2/3* mutants, suggesting that activation of the inhibitory D2-like pathway can antagonise DOP-1 receptors [102]. Interestingly, this study also displayed evidence that DOP-1 may be acting via the Gαq signalling pathway (encoded by *egl-30* and the RGS protein *eat-16*), rather than its usual Gαs coupling, which is also supported by previous studies [53].

Finally, in males, dopamine also promotes exploration essential for mate-search, even encouraging them to leave food sources. This behaviour increases the likelihood of finding mates, a primary goal for males. All five dopamine receptors (DOP-1, DOP-2, DOP-3, DOP-4 and LGC-53) contribute to high locomotor activity in males. Conversely, in hermaphrodites, dopamine promotes dwelling at food sources, maintaining low locomotor activity that conserves energy and ensures they remain in areas with sufficient resources for themselves and their progeny [103].

### 5.4. Food-dependent modulation of sensory responses

**Figure 5.**
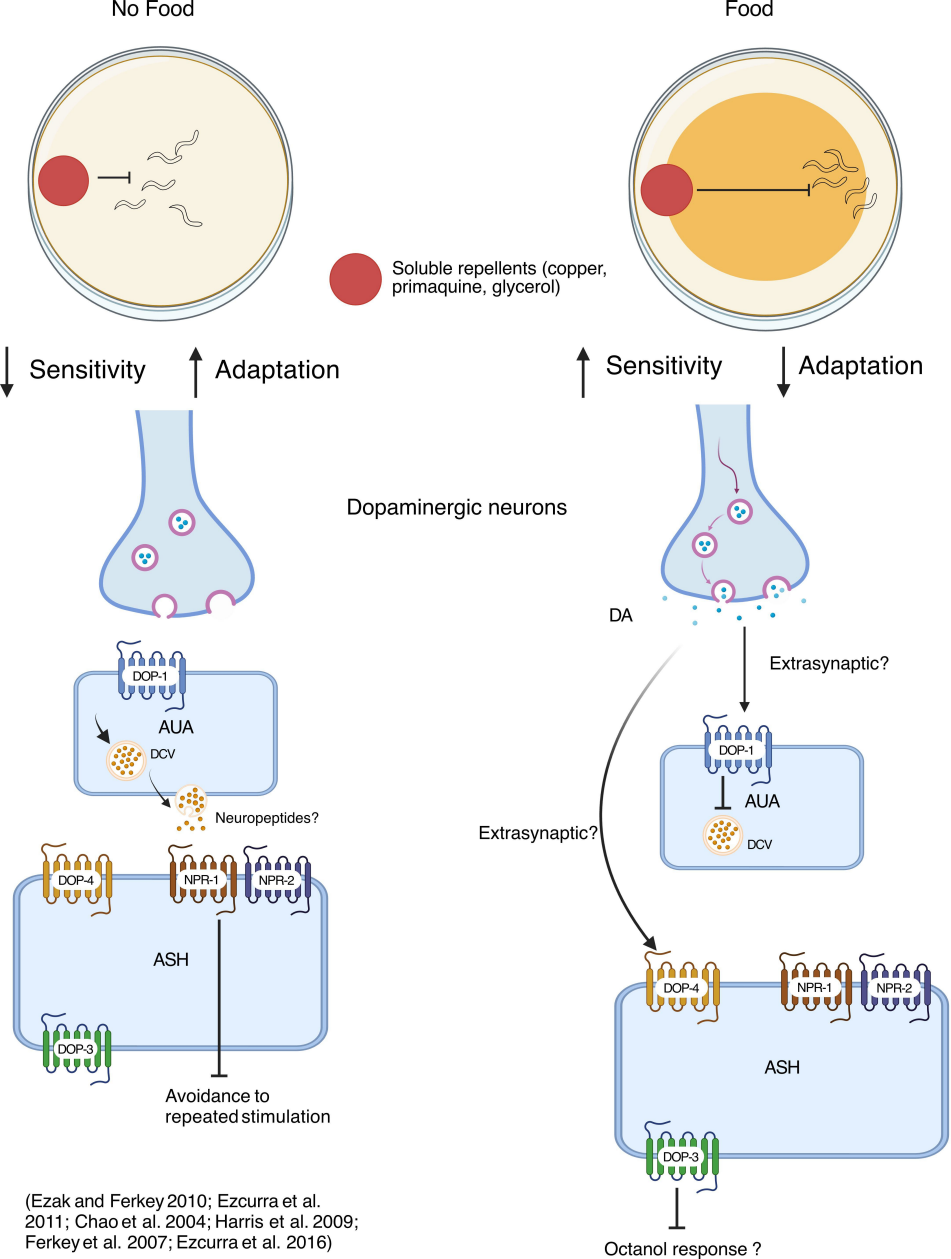
Dopamine mediates sensory responses in the presence of food. In the presence of food, dopamine enhances aversive responses to soluble repellents sensed by the ASH neuron, which expresses dopamine-activated GPCRs. By contrast, in the absence of food, this aversion is reduced, and adaptation occurs more rapidly, an effect mediated by the inhibitory action of neuropeptide receptors expressed in ASH. Additionally, the AUA interneuron regulates ASH adaptation extrasynaptically. Created in BioRender. Muralidhara, I. (2025) (https://BioRender.com/3ji3obg).

The presence of food, signalled internally by increased dopamine levels, modulates a wide range of sensory behaviours in *C. elegans*, altering the animal’s sensitivity to a wide range of stimuli. Most well studied is the effect of food on chemosensory behaviours, in particular, through the multimodal sensory neuron ASH. In the presence of food, *C. elegans* has been shown to exhibit significantly stronger avoidance of the soluble repellents copper, primaquine and glycerol, all sensed by ASH (figure 5; [104]). This is thought to be owing at least in part to the increased sensitivity of the nociceptor neuron ASH, via activation of the Gs-coupled DOP-4 receptor [104]. Interestingly, only the chemosensory not mechanosensory responses of ASH are affected by food availability [104]. ASH also senses the aversive odour octanol; here too, food enhances responses, however, this is thought to be mediated by serotonin [105,106]. Conversely, exogenous dopamine in fact reduces responsiveness to octanol via activation of the inhibitory DOP-3 receptor expressed in ASH (figure 5; [66]). In addition, *cat-2* mutant animals also display a hypersensitivity to octanol, suggesting that dopamine normally acts to reduce ASH sensitivity to octanol in the presence of food [107]. In either case, ASH does not directly receive synapses from the dopaminergic neurons, suggesting extrasynaptic mechanisms. While these may be direct via dopamine receptors expressed in ASH, an alternative pathway mediated through neuropeptide signalling via the neuropeptide receptor, NPR-1, has been suggested [108]. In this case, dopamine is thought to act on DOP-1 receptors in AUA interneurons, negatively regulating its neuropeptide release, leading to decreased activity in ASH. Without food, reduced dopamine signalling allows neuropeptide release from AUA, activating NPR-1 and NPR-2 in ASH (figure 5; [108]).

Dopamine also alters the worm’s response to other sensory inputs such as touch, CO_2_ concentration and temperature. When *C. elegans* is repeatedly exposed to a touch stimulus, it gradually habituates and ceases to perform avoidance behaviour in the form of reversals. The presence of food prolongs the duration of responses and reduces the rate of habituation [89,109]. This is thought to occur through the food-induced activation of dopaminergic CEP neurons, probably via synaptic connections from the touch receptor neurons ALM and AVM. The released dopamine, in turn, acts extrasynaptically on DOP-1 receptors located in ALM and AVM, thereby preserving their responsiveness to repeated touch [89]. Dopamine also influences CO_2_ response valence based on feeding state. Well-fed adults avoid CO_2_ while starved adults are attracted to it. While food is present, dopamine enhances excitatory responses of RIG neurons to CO_2_ and promotes inhibitory responses in AIY, promoting avoidance. Meanwhile, during starvation, octopamine promotes CO_2_ attraction by exciting AIY neurons. This mechanism allows worms to dynamically adjust their perception of CO_2_, a cue indicating both potential food (bacteria) and dangers—based on their hunger state [110]. In a similar paradigm, while food is present, animals experiencing the aversive condition of high temperature (25°C) continue to prioritise feeding. This behaviour is thought to be brought about by dopamine release that suppresses exploratory behaviour and promotes dwelling [111].

## 6. Learning

Across the animal kingdom, dopamine is well known for its involvement in learning, and here too, *C. elegans* has proved a valuable model organism in which to study the genetic and molecular mechanisms that govern the dopaminergic control of learning behaviours. Here, we briefly cover the major findings in the field, however, owing to the complexity and breadth of research in this area, we do not explore each area in detail. Several other review articles deal specifically with this task [112–116].

### 6.1. Non-associative learning

As described previously, upon repeated stimulation without consequence, *C. elegans* modulate their responses to aversive stimuli such as the touch response through a process known as habituation. A behavioural response to touch or vibration can be elicited by either gentle touch, stimulation of the anterior or posterior half of the worm with an eyelash pick or through tapping of the Petri dish to generate non-localized stimulation via vibration. In wild-type animals, the size of the response to tap decreases with repeated stimulations to approximately 20–40% after 30 taps [117]. Dopamine has been shown to be involved in this behaviour in a variety of ways. Not only are dopamine receptors broadly expressed in mechanosensory neurons (table 4), the dopaminergic neurons CEP and PDE also receive synaptic input from the touch neurons ALM and PLM. Animals lacking *dop-1* and *cat-2* exhibit faster habituation to tap stimulation [42,89], which is thought to act via a pathway including EGL-30 (Gαq), EGL-8 (PLC-β) and PKC-1 in the touch receptor neurons [89]. Meanwhile, *dat-1* mutants, which cannot reuptake dopamine, habituated more slowly than wild-type to tap stimuli [89]. Additional studies have found that habituation to anterior touch is also mediated by dopamine, in this case shown to act via the inhibitory DOP-2 receptor and its Gαi/o subunit, GPA-14, with mutants of *dop-2* and *gpa-14* habituating faster than wild-type animals [118]. DOP-2 is expressed in the dopaminergic ADE neurons, suggesting that dopamine acting via DOP-2 may downregulate dopamine release from ADE during repeated stimulations. As previously mentioned, this habituation is also modulated by the presence of food, which decreases the habituation rate.

Repeated activation of the nociceptive neurons ASH also leads to a decrease in response size consistent with habituation [104]. Animals lacking dopamine owing to a mutation in *cat-2* show significantly faster habituation to repeated optogenetic stimulation of ASH and repeated exposure to natural aversive cues sensed by ASH including CuCl_2_ [104,109]. In addition, animals in the presence of food also habituated faster [104,109], again pointing at the involvement of dopamine in the regulation of this behaviour. Of the GPCR dopamine receptors tested, only animals lacking the D1-like DOP-4 receptor showed a similar increased habituation to repeated optogenetic activation of ASH as *cat-2* mutants [109]. In contrast to DOP-1, DOP-4’s effect on ASH habituation appears to occur downstream of ASH excitation rather than within the ASH neuron itself [109].

*Caenorhabditis elegans* also show adaptation to other non-mechanical stimuli including chemical odours. For example, pre-treatment with a high dose of the attractive odour benzaldehyde leads to a reduction in its attractiveness on subsequent exposure [119]. While *cat-2* mutants act like wild-type in this behaviour, they are not affected by pretreatment with ethanol, which in wild-type animals enhances the adaptation response [120].

### 6.2. Associative learning

Despite their simple nervous system, *C. elegans* are capable of associative learning, measured through a range of behavioural paradigms. In one of these, worms are conditioned in a T-shaped maze, where food is present in one of the arms for five trials and then tested for a further five trials without a food cue. In the latter five test trials, wild-type animals are more likely to choose the side that previously contained the food. However, despite acting like wild-type in the initial conditioning phase, *cat-2* mutant animals are significantly less likely to choose the correct food location in the test phase [121]. Subsequent studies found that animals lacking *dop-3* are defective in both the initial food sensation and recall in the T-maze [122].

**Figure 6.**
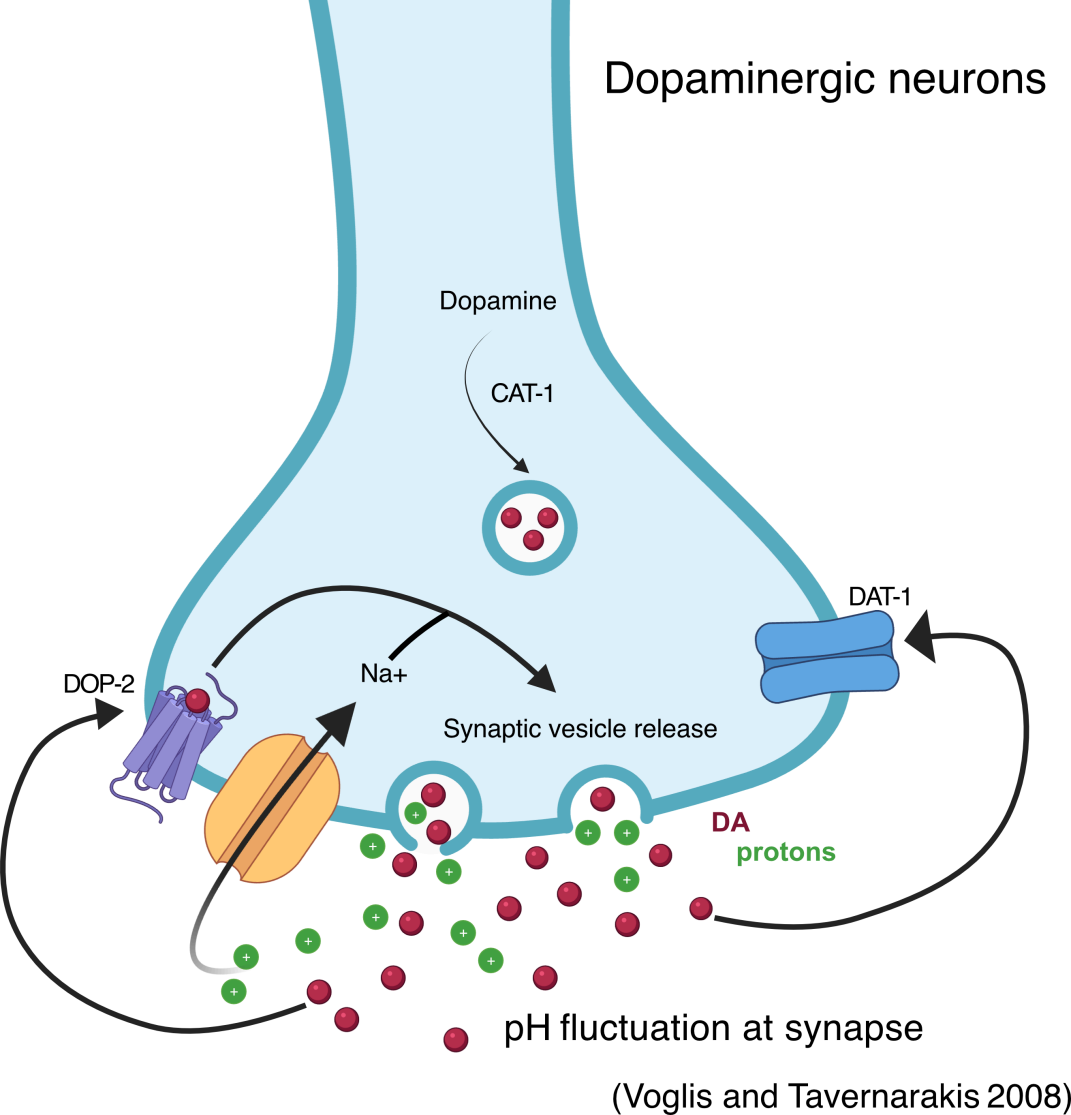
A positive feedback loop enhances dopaminergic signalling during associative learning. Protons released from synaptic vesicles lead to a change in local synaptic pH and activation of ASIC-1 (proton-activated DEG/ENaC channel) expressed in the dopaminergic neurons; this, in turn, leads to increased synaptic dopamine release. Created in BioRender. Muralidhara, I. (2025) (https://BioRender.com/3ji3obg).

In appetitive and aversive learning, conditioning animals in the presence or absence of food paired with an odour (or other stimulus) can lead to an association between these stimuli and result in an altered preference for the odour in a subsequent trial. In *C. elegans,* this can be achieved with a variety of stimuli including butanone, isoamyl alcohol, salt, temperature and mitochondrial stress [123–125]. Pairing isoamyl alcohol (an attractive odour) with the absence of food leads to a subsequent reduction in the attractiveness of isoamyl alcohol in wild-type animals. This effect is significantly reduced in *cat-2* mutants and can be rescued by the addition of exogenous dopamine, with mutants of *dop-1* and *dop-2*, but not *dop-3,* also showing defects in this behaviour [123]. Through a series of experiments, Voglis and colleagues proposed that ASIC-1 (a proton-activated DEG/ENaC channel), expressed in the dopaminergic neurons, establishes a positive feedback loop that reinforces dopamine release during learning (figure 6; [123]). In another example of associative learning, worms avoid low concentrations of NaCl only after conditioning with low NaCl in the absence of food; here too, *cat-2* mutants are defective in learning, which could be rescued by the addition of exogenous dopamine, as well as defects in *dat-1*, *dop-1*, *dop-2* and *dop-3* mutants [124]. Finally, dopamine has also been shown to be involved in ethanol preference following pre-exposure, as evidenced by association deficits in *cat-2* loss-of-function mutants [126] as well as in the learnt avoidance of bacteria when paired with mitochondrial stress [125].

### 6.3. Dopamine in memory and forgetting

Current research presents contrasting perspectives on dopamine’s precise function in memory processing. In a recent study, McMillen *et al.* propose that dopamine facilitates active forgetting, with lower dopamine levels in *cat-2* mutants extending memory retention through reduced forgetting mediated by D2-like receptors (DOP-2 and DOP-3) and requiring dopamine release from all dopaminergic neurons [127]. Conversely, a separate study by Raj and Thekkuveettil suggests that dopamine is critical for adaptive memory formation and recall, demonstrating that *cat-2* mutants exhibit both learning defects and accelerated forgetting [128]. This apparent contradiction may be explained by methodological differences in the conditioning paradigms used, in particular plate-based versus liquid assays, given that dopamine also modulates swimming behaviour, SWIP and swim-to-crawl transitions. These differences highlight how dopamine’s effects on memory may depend on specific behavioural contexts during learning acquisition. Despite these opposing findings on dopamine’s exact role in memory processing, both research perspectives converge on a fundamental conclusion: dopamine and its receptors are critically involved in regulating short-term olfactory associative memory in *C. elegans*.

## 7. Reproduction, sexual dimorphism and mating behaviours

Mating behaviour in *C. elegans* represents a complex, innate process driven by internal motivation and external cues. This behaviour portrays distinct phases including mate-finding, vulva location, spicule insertion and ejaculation, all regulated by a sexually dimorphic nervous system [129]. The male *C. elegans* nervous system contains 387 neurons, approximately 30% more than the hermaphrodite’s 302 neurons, with most male-specific neurons concentrated in the tail region forming interconnected groups associated with copulatory structures [130,131].

### 7.1. Sexual dimorphism in dopaminergic neurons

**Figure 7.**
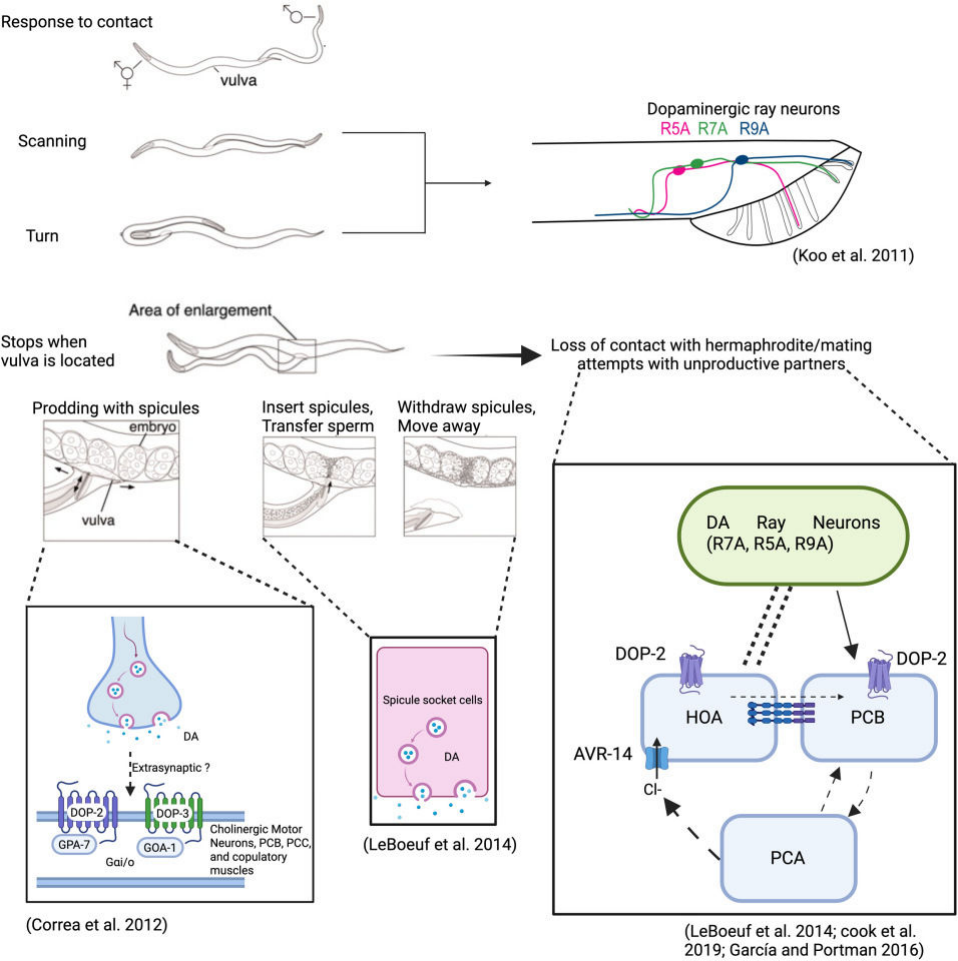
Overview of dopamine’s role in modulating male mating behaviour in *C. elegans*. Dopaminergic ray neurons (R5A, R7A and R9A) regulate scanning and turning behaviours that enable males to locate the hermaphrodite’s vulva. Dopamine also contributes to successful copulation by guiding spicule orientation and insertion into the vulva. During mating, if the male loses contact with the hermaphrodite or engages with unproductive partners, neurons such as HOA and PCB, together with dopamine signalling, help regulate corrective responses. Finally, dopamine released from the spicule socket cells is required for sperm release and plays a key role in establishing the post-ejaculatory refractory period. Partly reproduced from Jarrell *et al.* [132] with permission from AAAS. Created in BioRender. Muralidhara, I. (2025) (https://BioRender.com/3ji3obg).

While hermaphrodites have eight dopaminergic neurons (four bilateral pairs: CEPVL/R, CEPDL/R, ADEL/R and PDEL/R), males have six additional dopaminergic neurons in the tail. These male-specific dopaminergic neurons (R5A, R7A and R9A) line the dorsal ridge of the tail, sending projections along this ridge and extending into the sensory rays, where they play crucial roles in mating behaviour (figure 7; [11,133]). The establishment of this sexually dimorphic dopaminergic pattern involves several transcription factors. The ETS-domain transcription factor *ast‐1* serves as a phylogenetically conserved activator of dopamine biosynthesis gene transcription in both sexes. However, its activity is also regulated by sex-specific factors, particularly DM domain genes *dmd-3* and *mab-23,* which generally repress *ast‐1* activity in circuit sensory neurons, promoting acetylcholine fate over dopamine. However, in specific neurons (R5A, R7A and R9A), a TGFβ-family signalling pathway involving DBL-1 and the Hox gene *egl-5* blocks *dmd-3* and *mab-23* function, allowing *ast‐1* to promote dopaminergic fate. This intricate regulatory network ensures proper restriction of dopaminergic identity in male tail neurons [14,134–136].

### 7.2. Mating behaviours

As discussed previously, dopamine promotes high locomotor activity states in males (dispersal), enabling exploration of larger areas by encouraging them to leave bacterial lawns and thereby increasing the probability of them encountering potential mates [103]. Once a hermaphrodite has been found, males search or ‘scan’ for the vulva moving backwards along the body of the hermaphrodite with their tail fan, making a series of reproducible turns at the head and tail ends [131], thought to be controlled by the A-type ray neurons, which includes the dopaminergic R5A, R7A and R9A (figure 7; [137]). This turning behaviour is disrupted in dopamine-deficient *cat-2* males, which tend to make large, imprecise arcs instead of the sharp coil-like turns seen in wild-type animals [138].

Dopamine also plays a role in precise motor control during spicule insertion and copulation by antagonizing cholinergic mating circuits responsible for vulva location, spicule protraction and intromission. While cholinergic neurons (including neurons PCB and PCC) promote spicule insertion attempts, dopamine directs these attempts specifically towards the hermaphrodite’s vulva by dampening spurious, stimulus-independent sex muscle contractions. Without proper dopaminergic signalling, sex muscles contract inappropriately, and spicule insertions are not properly oriented. This antagonism operates through D2-like dopaminergic receptors DOP-2 and DOP-3, which function via Gαi/o proteins (GOA-1 and GPA-7) to inhibit signalling in cholinergic neurons and copulation musculature (figure 7; [139]). When the male loses contact with the hermaphrodite, dopamine released from R5A, R7A and R9A causes a reduction in activity of the synaptically connected HOA, PCA, PCB and sex muscles [140]. This increased activity of the dopaminergic ray neurons correlates with males adopting a ventrally arched posture after prolonged prodding. DOP-2 signalling then enhances UNC-7 gap junctions between HOA and PCB, allowing inhibitory signals mediated by the AVR-14 glutamate-gated chloride channel in HOA to pass into PCB, reducing its excitability. This dampening effect also occurs during mating attempts with unproductive partners. During prolonged, unsuccessful spicule insertion attempts, the activity of the PCA neurons increases until it eventually leads to hyperpolarization of HOA neurons and activation of the dopaminergic ray neurons. The inhibition mediated via this dopamine release increases the probability of the male disengaging to seek a more receptive mate (figure 7; [32,141]).

Finally, dopamine released from the spicule socket cells is essential for sperm release and contributes to the post-ejaculatory refractory period, combining successful ejaculation with reduced mating drive. By promoting ejaculation and immediately reducing activity levels, dopamine enforces a delay between matings, potentially increasing the likelihood of successive matings with different hermaphrodites (figure 7; [32]).

### 7.3. Egg laying

Egg-laying behaviour in *C. elegans* is regulated through a complex neural network involving multiple neurotransmitters including ACh, serotonin, dopamine and octopamine. Animals increase their egg-laying rate in the presence of food and exogenous serotonin [98]. By contrast, exogenously applied dopamine has been shown to inhibit egg laying in wild-type worms in the presence of food or exogenous serotonin [142,143]. Early experiments also described a rescue effect of D2 antagonists on gain-of-function mutants of *elg-2* (potassium channel) which are defective in egg laying [143]. The inhibitory effect of dopamine on egg-laying rate in the presence of serotonin requires MOD-1 (serotonin-gated chloride channel; [144]), and FAHD-1 (a mitochondrial oxaloacatate decarboxylase; [145]), although the molecular mechanisms for these interactions are not known.

**Figure 8.**
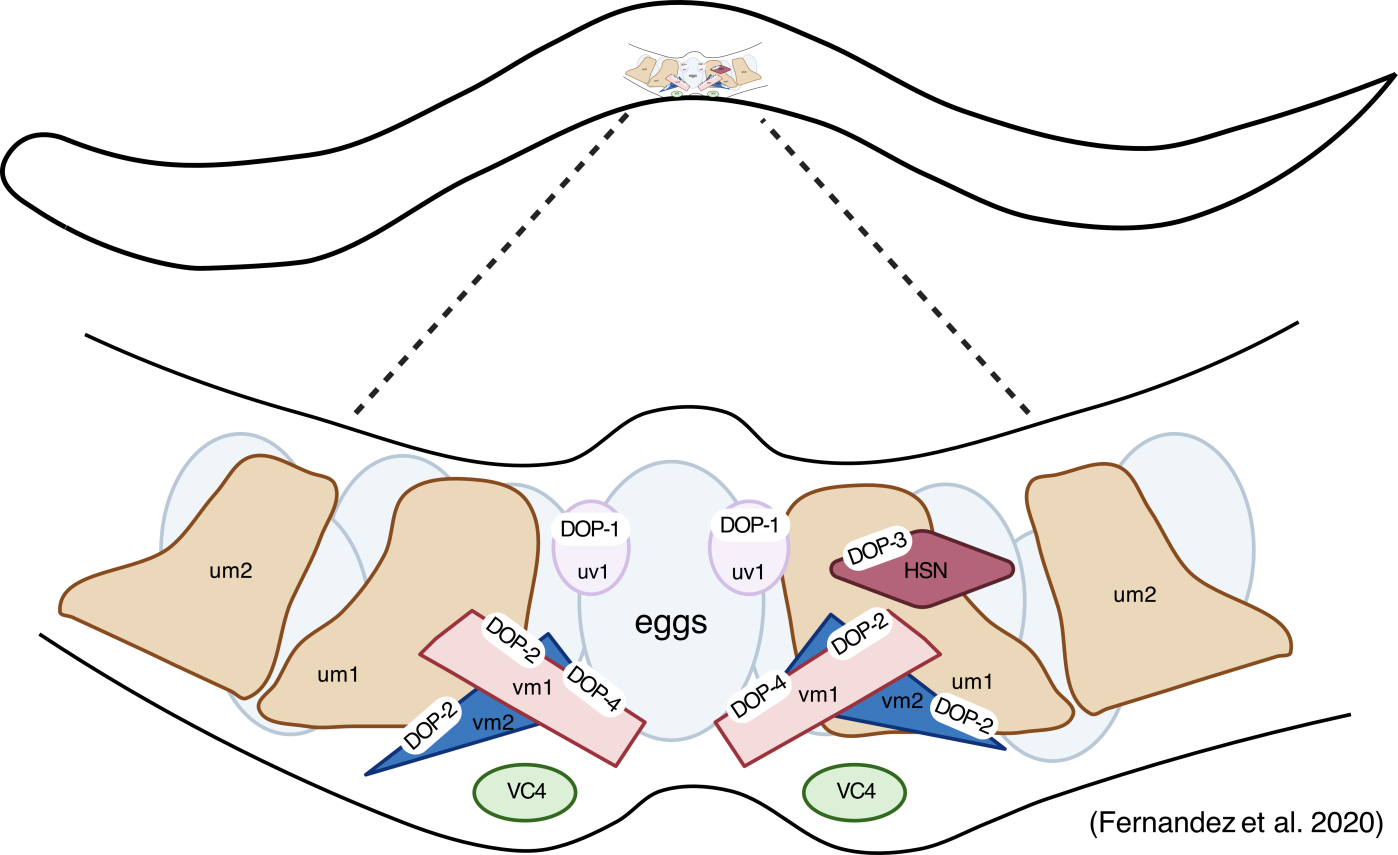
Dopamine-activated GPCR expression across the egg-laying circuit. Using expression data from [69]. Created in BioRender. Muralidhara, I. (2025) (https://BioRender.com/3ji3obg).

Interestingly, subsequent studies found that in swimming animals, exogenous dopamine appeared to cause a modest increase in egg-laying behaviour, which is typically inhibited during swimming [146]. There is also evidence that endogenous dopamine can affect the developmental stage at which eggs are laid. Animals lacking *cat-2* have been shown to lay more developed eggs than wild-type [147], both *cat-2* loss-of-function mutants and *cat-2* overexpressors also show a slight decrease in eggs retained *in utero* [148]. To elucidate these complex and potentially contradictory roles for dopamine in egg-laying behaviour, fluorescent localization studies identified the localization of all four known dopamine GPCR receptors (DOP-1-4) within the egg-laying system. Each is expressed in specific cell types: DOP-1 in uv1s, DOP-2 in vm1/vm2, DOP-3 in HSNs and DOP-4 in vm1/vm2 (figure 8; [69]). Since no dopaminergic neurons directly synapse onto the egg-laying circuit, dopamine probably mediates its effects extrasynaptically. Fernandez and colleagues also sought to characterize the behavioural contribution of all monoamine-activated GPCRs in the egg-laying circuit and found that overexpression of *dop-2-4* but not *dop-1* led to a small but significant decrease in the number of unlaid eggs, while animals lacking *dop-4* also showed a similar reduction in unlaid eggs. Whether these effects represent an increase in egg-laying rate or a reduction in egg production remains unclear [69].

**Figure 9.**
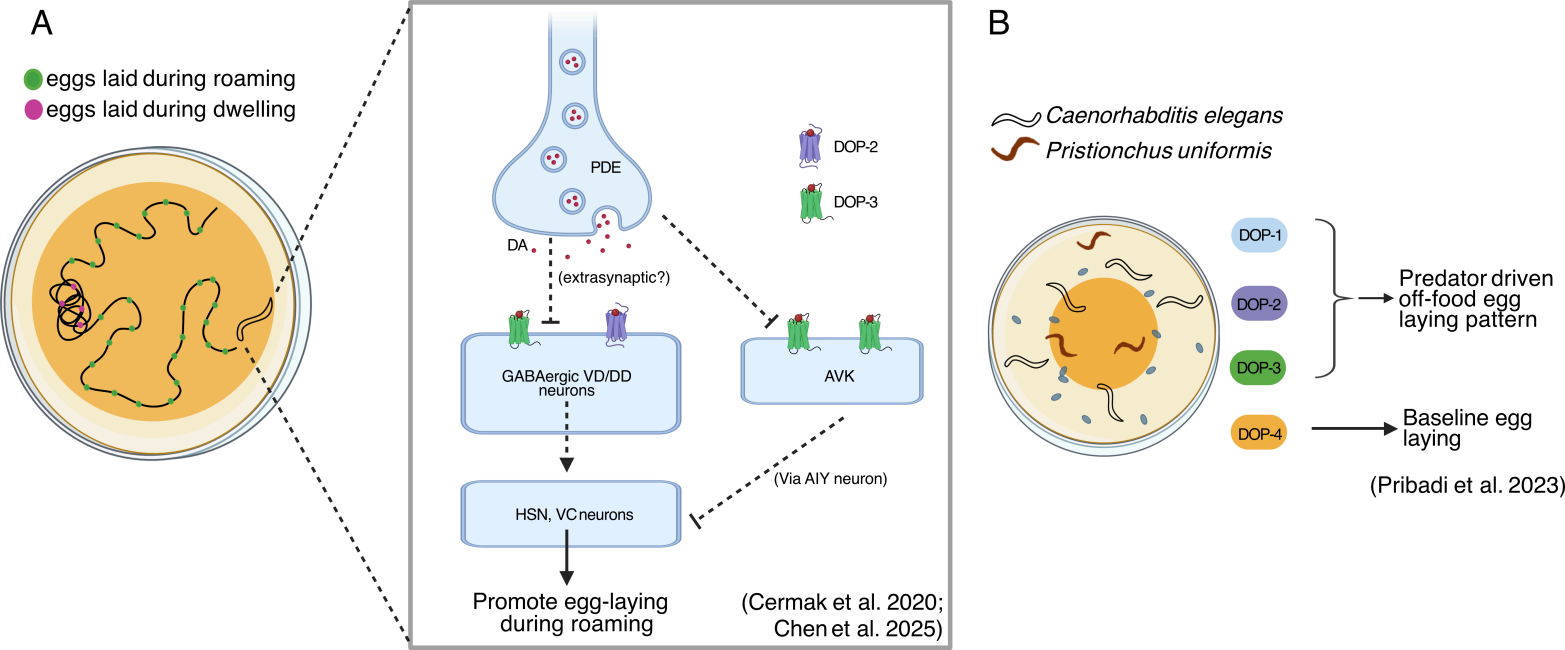
Mechanisms of dopamine-mediated control of egg-laying behaviours. (A) Dopamine released from PDE neuron gates food-dependent egg laying by disinhibiting HSN/VC activity via DOP-2, DOP-3 on VD/DD neurons and DOP-3 on AVK. (B) Dopamine shifts egg laying away from food, with DOP-1, DOP-2 and DOP-3 mediating predator-driven off-food egg-laying pattern and DOP-4 tuning baseline egg distribution. Created in BioRender. Muralidhara, I. (2025) (https://BioRender.com/3ji3obg).

Egg-laying rate and feeding states are closely aligned, with egg-laying frequency increasing while animals are in the feeding roaming state compared with dwelling state. It has been shown that dopamine, released from PDE neurons during high-speed roaming, acts through DOP-2 and DOP-3 receptors, potentially inhibiting GABAergic VD/DD neurons, thereby disinhibiting the egg-laying circuit involving HSN and VC neurons and promoting egg laying. This coupling mechanism enhances egg dispersal across food sources, benefiting progeny survival (figure 9A; [79]). Consistent with these findings, additional studies demonstrated that dopamine signalling is crucial for worms’ propensity to disperse eggs away from the main food source, a behaviour termed baseline egg laying. When predators are present alongside food, this behaviour is further enhanced, as worms strategically deposit eggs at the lawn’s edge and along streaks away from the central region—a response known as predator-mediated egg laying. Loss of dopamine synthesis or receptor function affects both the baseline probability of laying eggs off the lawn and can significantly attenuate the magnitude of the predator-induced increase in off-lawn egg laying. Notably, this avoidance strategy is triggered specifically by bites from the naturally occurring predator *Pristionchus uniformis* rather than chemosensory cues, suggesting a direct physical interaction detection mechanism. Receptor studies revealed differential contributions of dopamine receptors: combinations of DOP-1, DOP-2 and DOP-3 receptors are particularly critical for the predator-evoked change in egg-laying location, while DOP-4 appears to play a more specialized role in modulating the general baseline tendency for off-lawn egg deposition (figure 9B; [149]).

Dopamine also mediates food-dependent egg laying via AVK interneurons, which normally inhibit egg-laying behaviour. When food is present, dopamine released from food sensing PDE neurons acts on AVK interneurons, reducing their activity. This inhibitory action occurs through DOP-3, effectively disinhibiting the egg-laying circuit and coupling food presence to reproductive behaviour [150].

## 8. Discussion

A vast repertoire of data now supports the breadth and complexity of dopamine signalling in the nematode *C. elegans*; however, despite decades of research, new and old findings are still opening further areas for investigation. Perhaps surprisingly, considering the depth of knowledge of dopaminergic signalling in *C. elegans,* relatively little is truly known about the synthesis and metabolism of dopamine in the nematode. For most enzyme homologues, it remains unclear whether they truly function in the same capacity as their mammalian counterparts. This is especially true for the putative COMT encoding genes, *comt-1-4*, but also for the MAO pathways. It is also unclear which neurons, glia or other cells are involved in the metabolism of dopamine after its release. The dopaminergic neurons themselves express the DAT-1 reuptake transporter, suggesting that in some capacity these neurons play a role in dopamine recycling, probably via the MAO pathway. Given also that *cat-2* mutants still have approximately 40% of wild-type levels of dopamine and that an alternative pathway for serotonin biosynthesis has only recently been discovered [40], there may also be undescribed alternative dopamine synthesis pathways, possibly involving many of the uncharacterized putative aromatic amino acid decarboxylase encoding genes [37].

Another area with incomplete and conflicting characterization is the expression pattern and localization of dopamine receptors in *C. elegans*. As is clearly depicted in table 4, there are significant differences in expression pattern between reporter strains and RNAseq; for example, ion channel receptors appear to be expressed in dopaminergic neurons according to RNAseq but are not present in any of the published reporter strains, leaving the question of whether these receptors can act as presynaptic auto receptors unanswered. There is also a sparse description of the expression pattern of dopamine receptors in the male. It will be important to resolve these differences using CRISPR-generated endogenous reporters and more accurate cell identification tools [151]. By contrast, the behavioural characterization of the dopaminergic circuits, in particular dopamine-activated GPCRs in *C. elegans,* has been broad and thorough. However, the contribution of dopamine-gated ion channels to behaviour has been relatively little studied to date, raising the question of whether these receptors function as true dopamine receptors *in vivo*. In addition, the still sparse characterization of the over 100 LGIC genes in *C. elegans* suggests that further dopamine receptors may yet be discovered.

Looking beyond *C. elegans* as a model, the dopaminergic systems of various other invertebrates have also been extensively studied, in particular that of *Drosophila* in which, much like in nematodes and mammals, dopamine has been shown to be involved in a broad range of behaviours including locomotion, learning and sensory processing [152–155], many of these roles for dopamine have also been identified in honeybees [156]. Interestingly, however, recent identification of a dopamine-gated ion channel in bees but not flies [60] suggests that despite their close evolutionary relationship and similarities in brain structures, the molecular mechanisms that govern dopamine signalling in these species may diverge. In molluscs too, dopamine has been shown to be involved in a breadth of behaviours including locomotion and learning [157]; here too dopamine-activated GPCRs and ion channels are present. With ever-advancing molecular tool kits available for a broader selection of animal species, it will be interesting to understand in more detail the evolutionary processes that have shaped dopamine signalling in the nervous systems of diverse animals.

Finally, a major discussion in dopamine signalling in general is the spatio-temporal dynamics of dopamine signals. Largely owing to the type of dopamine receptors present in mammalian systems (GPCRs), their apparent subcellular localization [158], and the expression pattern of receptors in model organisms including *C. elegans* [56], it has long been assumed that dopamine acts via slow volume transmission often acting on receptors over considerable distances. However, over the last decade, evidence for near synaptic or synaptic transmission at much faster time scales and over shorter distances has been building [57]. This includes evidence that mammalian dopaminergic neurons, despite co-releasing other neurotransmitters, have specific dopamine release sites that can be triggered by action potentials (albeit only representing a small fraction of total dopamine release) [159,160], as well as peri-synaptic localization of receptors [161]. Even in *C. elegans,* the behavioural conditions that lead to the activation of dopaminergic neurons and their subsequent release dynamics are not well characterized and usually inferred from behavioural experiments. For example, only two of the six CEP neurons have been shown through calcium imaging to be activated by food entry [90]. Large-scale studies using calcium imaging to determine functional connectivity between neurons also paint a complex picture of dopaminergic signalling with strongly connected pre- and post-synaptic neurons not always displaying functional connectivity, and non-synaptically connected neurons showing strong correlation [162,163]. This points at a combination of both synaptic and extrasynaptic dopaminergic signalling, which may be acting at time and spatial scales not easily measured with whole cell calcium imaging. In addition, the identification of multiple dopamine-gated ion channels from the pentameric cys-loop family, which includes classic synaptic receptors such as GABA_A_R and nAChRs, not only in nematodes but across invertebrates also suggests that faster acting dopamine transmission may be possible. Over the coming years, it will be interesting to see how advanced super resolution microscopy, genetically encoded dopamine sensors [164,165] and electrophysiology will support or reject the notion of fast dopamine transmission across animals.

## Data Availability

This article has no additional data.
